# Nutritional status of young children in Mumbai slums: a follow-up anthropometric study

**DOI:** 10.1186/1475-2891-11-100

**Published:** 2012-11-23

**Authors:** Sushmita Das, Ujwala Bapat, Neena Shah More, Glyn Alcock, Armida Fernandez, David Osrin

**Affiliations:** 1 Society for Nutrition, Education and Health Action (SNEHA), Urban Health Centre, Chota Sion Hospital, 60 Feet Road, Shahunagar, Dharavi, Mumbai, 400017, Maharashtra, India; 2 Institute for Global Health, UCL Institute of Child Health, 30 Guilford Street, London, WC1N 1EH, UK

## Abstract

**Background:**

Chronic childhood malnutrition remains common in India. As part of an initiative to improve maternal and child health in urban slums, we collected anthropometric data from a sample of children followed up from birth. We described the proportions of underweight, stunting, and wasting in young children, and examined their relationships with age.

**Methods:**

We used two linked datasets: one based on institutional birth weight records for 17 318 infants, collected prospectively, and one based on follow-up of a subsample of 1941 children under five, collected in early 2010.

**Results:**

Mean birth weight was 2736 g (SD 530 g), with a low birth weight (<2500 g) proportion of 22%. 21% of infants had low weight for age standard deviation (z) scores at birth (<−2 SD). At follow-up, 35% of young children had low weight for age, 17% low weight for height, and 47% low height for age. Downward change in weight for age was greater in children who had been born with higher z scores.

**Discussion:**

Our data support the idea that much of growth faltering was explained by faltering in height for age, rather than by wasting. Stunting appeared to be established early and the subsequent decline in height for age was limited. Our findings suggest a focus on a younger age-group than the children over the age of three who are prioritized by existing support systems.

**Funding:**

The trial during which the birth weight data were collected was funded by the ICICI Foundation for Inclusive Growth (Centre for Child Health and Nutrition), and The Wellcome Trust (081052/Z/06/Z). Subsequent collection, analysis and development of the manuscript was funded by a Wellcome Trust Strategic Award: Population Science of Maternal and Child Survival (085417ma/Z/08/Z). D Osrin is funded by The Wellcome Trust (091561/Z/10/Z).

## Background

Severe acute malnutrition in childhood has become steadily less common in India [1]. Malnutrition remains ubiquitous, however, with worrying implications for both short-term survival and longer-term wellbeing, economic growth, and socioeconomic inequalities [2]. An estimated 52 million children are stunted (height for age standard deviation [z] score < −2) [3]. Urban levels of childhood malnutrition are lower than rural, but the most recent National Family Health Survey (NFHS-3: 2005–6) described stunting in 40%, wasting (weight for height z score < −2 SD) in 17%, and low weight for age (<−2 SD) in 33% of urban children under 5 [4]. In the same survey, 47% of children from Mumbai slum areas were stunted, 16% wasted, and 36% had low weight for age [5]. Why this should be remains unresolved [6], although trans-generational, environmental, and dietary factors probably all play a part. There are questions about the underlying dynamics of nutrition in the face of substantial increases in gross national income per capita [1], and concerns about inequalities [7].

Ideas about the development of childhood malnutrition are also changing. Of critical importance is the window of vulnerability within which interventions may be effective. It has long been known that growth trajectories are set early in life, but recent work on the developmental origins of health and disease has focused attention on gestation and the first two years (‘the 1000 days’: see, for example, http://www.thousanddays.org). A second stimulus to rethinking has been the switch to classification using the World Health Organization (WHO) standards of 2006 (http://www.who.int/childgrowth/standards/en/). In a 2001 analysis of 39 national samples against National Center for Health Statistics (NCHS) standards, height for age declined until 24 months and then stabilized, weight for height declined until 15 months, and weight for age faltered rapidly from three to 12 months, followed by some catch-up [8]. However, a recent analysis of data from 54 countries, using the WHO standards, suggested that early growth faltering was more pronounced and that the window of opportunity included pregnancy and the first 24 months [9]. The authors pointed to “… a much greater problem of undernutrition during the first 6 months of life than previously believed, bringing coherence between the rates of undernutrition observed in young infants and the prevalence of low birth weight and early abandonment of exclusive breastfeeding” [9].

The Society for Nutrition, Education and Health Action (http://www.snehamumbai.org) works to improve the health of women and children in slums in Mumbai, Maharashtra. Even as newborn survival increases, we have become increasingly concerned about the parlous state of child nutrition. Data collection for a large cluster randomized controlled trial – which did not focus on nutrition - included information on birth weight transcribed from institutional birth records. Involving 24 intervention and 24 control settlements and a total population of ~283 000, the trial tested the effects of community mobilization through local women’s groups on perinatal care and mortality [10]. We found no differences between trial arms in uptake of antenatal care, reported work, rest, and diet in later pregnancy, institutional delivery, early and exclusive breastfeeding, or care-seeking [11].

Because a surveillance system was in place, we followed up a sample of infants from the dataset to understand major nutritional concerns. We had three objectives: to describe the proportions of underweight, stunting, and wasting in young children in urban slums, to test the hypothesis that underweight (low weight for age) is largely explained by low height for age, and to examine the relationships between z scores and age.

## Methods

### Setting

Mumbai is India’s most populous city, a base for commerce and the film industry. The city generates 33% of national tax collection, 40% of share in foreign trade, and 60% of customs duty [12]. Recent census figures estimate Mumbai’s population at about 12.5 million, with a sex ratio of 838 females per 1000 males and a female literacy rate of 86% [13]. Livelihood expansion has been largely in the informal and low-wage sectors, with increasing self-employment and feminization of the workforce [12]. Slums are home to at least half of the population. In 2002, a United Nations expert group recommended a provisional operational definition of slums based on multiple domains: inadequate access to safe water, sanitation and other infrastructure, poor structural quality of housing, overcrowding, and insecure residential status [14]. This is the general framework we use, although the parameters of each of the components are not clearly defined.

### Data collection

We used two linked datasets for the analysis: a large primary dataset collected through prospective registration of births in 48 slum areas (each of at least 1000 households), and follow-up of a subsample of children. The primary dataset accrued during the trial of community mobilization in 48 urban slum areas [10]. A cadre of 99 local women identified births, infant and child deaths, and maternal deaths. These were confirmed by 12 interviewers, who visited homes, explained the study, requested verbal consent, and interviewed mothers using a predominantly closed questionnaire at about six weeks after delivery. The interview included questions on demography, birth history, assets, education, and infant feeding. We did not measure birth weight: mothers were asked if they had institutional record cards for their deliveries, and birth weights were copied from them.

Data collection for a baseline year and the trial itself ran from October 2005 to September 2009. Data were collected for the follow-up study from January to August 2010. From surveillance records, we extracted a list of children born from October 2005 to March 2009 (such that the youngest children would be around 6 months of age, and the oldest around 58 months). Each was assigned a random number generated in Microsoft Excel 2007 (Microsoft Corporation). We blocked the list by cluster, aiming for an unweighted sample of 40 children per cluster. Because we wanted a spread of ages, we divided the list of possible participants into four blocks, one for each of the four years of original data collection. Each interviewer was given a list of 40 children (4 blocks of the first 10 sorted random numbers within each block). She attempted to locate the children, with the instruction that she should continue until she had found 40. If she reached the end of a list without reaching the target, she was given a new one. This was repeated 3 times to enrol the required number of children. Interviewers did not have to enrol 10 children in each band.

When an interviewer found a mother at home, she explained the study and requested signed consent for participation. Children <2 years were weighed on an Equinox BE-EQ44 digital infant scale accurate to 10 g (http://www.equinox-india.com), children 2 and over on an Avon Corporation digital scale (http://www.avon.co.in), and their lengths and heights measured with Bio Plus stadiometers. Data were entered in Microsoft Access 2003 (Microsoft Corporation). Information volunteered by participants remained confidential, and names and addresses were deleted from the datasets before analysis. With 17 000 birth weight measurements, an estimate of low birth weight prevalence of 23-25% would have a precision of less than 1% (at 95% confidence level). With a sample of 1500 children at follow-up, estimates of the proportions with weight or height for age less than two z scores below the median (of 35% and 45%, respectively) would have a precision of 2-3%.

### Statistical analysis

Analyses were done in Stata 12 (Stata Corporation, College Station, USA). We summarized findings from both datasets with frequencies and proportions for categorical variables, and means and standard deviations (SD) for continuous variables. We generated z scores for weight for age, height for age, and weight for height, using the 2006 WHO growth standards and the *zscore06* module [15]. Asset scores were derived from standardized weights for the first component of a principal components analysis [16,17]. We used regression models for a descriptive analysis of associations of potential determinants with birth weight z scores (linear models for continuous and logistic models for binary dependent variables). The models included random effects for cluster. The inclusion of covariates for potential confounders was based on a conceptual framework with four levels. At *household* level, we tested asset score as a continuous variable, nuclear family as a binary variable, religion as a set of four dummy variables, and trial allocation group as a binary variable. At *maternal* level, we tested age as a continuous variable and Indian levels of schooling as dummy variables. At *pregnancy* level, we tested a binary variable describing three or more antenatal care episodes because this was a local recommendation and almost all mothers had made at least one visit. At *newborn infant* level, we tested birth order as an ordered categorical variable, and sex, twin status and preterm delivery as binary variables. We examined associations of each these potential confounders with birth weight z score and proportion of underweight (< −2 z scores) in univariable models, and then in multivariable models within each level. Adjusted models included covariates with associations at p < 0.1: asset score, religion, maternal age and schooling, three or more antenatal care visits, birth order, twin status and preterm. Forcing the trial allocation covariate into the model did not improve fit. Individual z scores were scatter-plotted against age. We used linear models on the basis of a fractional polynomial comparison, and fitted regression lines with 95% confidence intervals. In modelling change in weight for age z score with age, we treated the data as time series and included a fixed effect to address repeated measures within children.

### Ethical statement

Cluster-level and individual verbal consent for the primary study were given after community meetings with general practitioners, community based organizations, non-government organizations, municipal representatives, political officers of major parties, and social workers. Ethical permission was granted by the Independent Ethics Committee for Research on Human Subjects (Mumbai, reference IEC/06/31).

### Role of the funding source

The sponsors had no role in the study design, data collection, analysis, interpretation or writing of the article. DO had access to all study data and responsibility for the decision to submit for publication.

## Results

**Figure 1:**
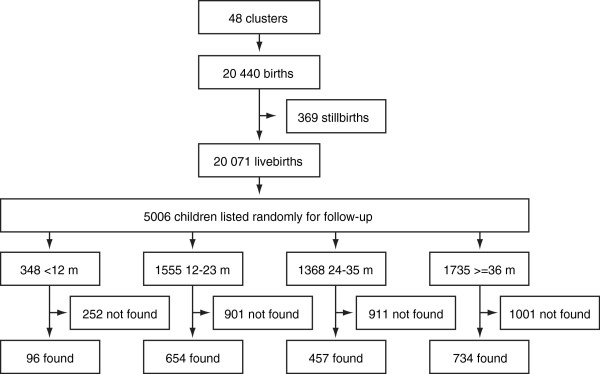
Study profile.

Figure 1 summarizes recruitment. 1941 children were found at follow-up, a median 39 per cluster (interquartile range 25–56). The apparent unevenness of the age spread is the result of two factors. First, recruitment effectively began around 6 months of age, so the block of children under 12 months is small. Because no children had reached the age of 5 years, we collapsed infants over 3 years old into a single block, making it larger. Second, the turnover of slum residents is such that the families of younger children were less likely to have moved: there were more children aged 12–23 months than aged 24–35 months.

### Birth weight

Birth weights were available for 17 318 infants (86% of livebirths): 9173 males and 8145 females. Table 1 compares groups with birth weight available and unavailable. Since data were extracted from institutional records, they tended to be unavailable for the 2860 (14%) infants who were born at home. Institutional delivery increases with socioeconomic status, and individuals with missing data were poorer: mean asset score was lower and there were more families from lower asset quintiles. They were also less likely to have been to school and more likely to be of Muslim faith.

**Table 1:** Comparison of groups for whom birth weight was and was not available, and of mothers whose children were and were not followed up

	**Birth weight available**		**Birth weight unavailable**		**Followed up (data for mothers)**		**Not followed up (data for mothers)**	
Infants (%)	17318	(100)	3122	(100)	1631	(100)	18809	(100)
Institutional delivery (%)	16894	(98)	686	(22)				
Mean asset score (SD)	0.101	(0.983)	−0.561	(0.903)	0.185	(0.948)	−0.015	(1.002)
Asset quintile 1 (%)	2884	(17)	1203	(39)	208	(13)	3879	(21)
Asset quintile 2 (%)	3277	(19)	813	(26)	313	(19)	3777	(20)
Asset quintile 3 (%)	3549	(21)	549	(18)	348	(21)	3750	(20)
Asset quintile 4 (%)	3977	(22)	326	(10)	399	(24)	3904	(21)
Asset quintile 5 (%)	3631	(21)	231	(7)	363	(22)	3499	(19)
Maternal education								
No schooling (%)	3825	(22)	1592	(51)	355	(22)	5062	(27)
Primary (%)	944	(5)	221	(7)	85	(5)	1080	(6)
Secondary (%)	10740	(62)	1215	(39)	1040	(64)	10915	(58)
Higher (%)	1809	(11)	94	(3)	151	(9)	1752	(9)
Religion								
Hindu (%)	8583	(50)	1248	(40)	889	(54)	8942	(46)
Muslim (%)	7519	(43)	1787	(57)	597	(37)	8709	(48)
Other (%)	1216	(7)	87	(3)	145	(9)	1158	(6)

Mean birth weight was 2736 g (SD 530 g): 2776 g (538 g) for males and 2691 g (518 g) for females. 3868 newborn infants were low birth weight (<2500 g; 22.3%) and 211 were very low birth weight (<1500 g; 1.2%). Low birth weight was seen in 20.1% of males (1842/9173) and 24.9% of females (2026/8145). These figures are probably underestimates because of data heaping at 2500 g. 6.5% of infants were classified as preterm (1305/20 071). Of low birth weight infants, 16.5% were preterm (637/3868). Mean weight for age z score at birth was −1.31 (SD 1.26): -1.31 (1.25) for males and −1.32 (1.27) for females. 3599/17 318 infants had birth weight for age z scores below −2 (20.8%); for males, 19.8% (1821/9173), and for females, 21.8% (1778/8145).

### Associations with birth weight

Table 2 shows that, in adjusted models, weight for age z score at birth increased with socioeconomic status in terms of household asset score. Infants born to Muslim mothers had higher z scores than Hindu, and Buddhist infants lower. z scores also increased with maternal age, higher grades of maternal schooling, mothers having made three or more antenatal care visits, and mothers saying that they had rested and eaten more in later pregnancy than before pregnancy, and that they had taken more packets of iron supplements. Higher birth order was associated with higher weight for age z scores at birth, and twin pregnancies and preterm deliveries with lower. Similar associations were seen when the outcome was low weight at birth on the basis of a −2 z score cut-off, apart from an attenuation of the association with maternal age.

**Table 2:** Weight for age z scores at birth: associations with potential predictors in regression models

	**Weight for age z score at birth**				**Weight for age z score < −2 SD**			
**N = 17318**	**Univariable**		**Multivariable**		**Univariable**		**Multivariable**	
	**Coefficient**	**(95% CI)**	**Coefficient**	**(95% CI)**	**OR**	**(95% CI)**	**aOR**	**(95% CI)**
**Household**								
Allocation to trial intervention (reference: control)	−0.055	(−0.126, 0.017)	0.019	(−0.034, 0.071)	1.051	(0.960, 1.150)	0.963	(0.869, 1.066)
Asset score (continuous)	0.070	(0.050, 0.091)	0.074	(0.054, 0.094)	0.900	(0.865, 0.937)	0.867	(0.850, 0.927)
Nuclear family (reference: extended family)	0.000	(−0.008, 0.008)	0.000	(−0.008, 0.008)	0.992	(0.972, 1.012)	0.993	(0.973, 1.015)
Religion (reference: Hindu)								
Muslim	0.154	(0.107, 0.200)	0.144	(0.101, 0.187)	0.887	(0.816, 0.963)	0.867	(0.793, 0.949)
Buddhist	−0.223	(−0.317, 0.129)	−0.214	(−0.302, -0.125)	1.383	(1.176, 1.627)	1.375	(1.157, 1.634)
Other	0.105	(−0.033, 0.244)	0.081	(−0.051, 0.214)	0.933	(0.710, 1.225)	0.952	(0.715, 1.267)
**Mother**								
Age (continuous, in years)	0.020	(0.015, 0.024)	0.009	(0.004, 0.015)	0.981	(0.972, 0.990)	0.989	(0.978, 1.000)
Schooling (reference: none)								
Primary	−0.058	(−0.148, 0.032)	−0.033	(−0.012, 0.053)	1.145	(0.968, 1.364)	1.124	(0.940, 1.345)
Secondary	−0.030	(−0.077, 0.018)	0.006	(−0.041, 0.054)	1.038	(0.947, 1.139)	1.019	(0.921, 1.129)
Higher	0.140	(0.068, 0.211)	0.189	(0.116, 0.262)	0.839	(0.725, 0.970)	0.812	(0.691, 0.954)
**Pregnancy**								
3 or more antenatal care visits (reference: <3 visits)	0.209	(0.117, 0.301)	0.179	(0.054, 0.094)	0.687	(0.583, 0.809)	0.716	(0.602, 0.851)
Took more rest in third trimester than before pregnancy	−0.017	(−0.061, 0.026)	0.042	(0.001, 0.084)	0.957	(0.880, 1.042)	0.884	(0.808, 0.967)
Did less work in third trimester than before pregnancy	−0.054	(−0.096, -0.012)	0.016	(−0.024, 0.056)	1.038	(0.958, 1.125)	0.948	(0.870, 1.034)
Ate more food in second and third trimesters than before pregnancy	0.108	(0.056, 0.159)	0.130	(0.081, 0.179)	0.876	(0.790, 0.970)	0.833	(0.747, 0.928)
Packets of iron tablets taken (1, 2, 3; reference: none)	0.061	(0.042, 0.081)	0.043	(0.023, 0.063)	0.895	(0.863, 0.929)	0.921	(0.883, 0.959)
**Newborn infant**								
Birth order (reference: first baby)	0.115	(0.093, 0.137)	0.108	(0.082, 0.134)	0.900	(0.862, 0.941)	0.896	(0.848, 0.947)
Female infant (reference: male)	−0.011	(−0.049, 0.026)	−0.003	(−0.039, 0.033)	1.127	(1.047, 1.213)	1.123	(1.041, 1.212)
Twin (reference: singleton)	−1.724	(−1.865, -1.584)	−1.528	(−1.665, -1.391)	10.174	(7.911, 13.085)	9.182	(7.065, 11.933)
Preterm (reference: term)	−1.182	(−1.254, -1.110)	−1.077	(−1.148, -1.006)	4.513	(3.999, 5.093)	4.119	(3.637, 4.665)

Multivariable models include covariates for asset score, religion, maternal age and schooling, three or more antenatal care visits, birth order, twin status and preterm. CI: confidence interval. OR: odds ratio. aOR: adjusted odds ratio. SD: standard deviation.

### Anthropometry of children at follow-up

Birth weights were available for 1511 of the 1941 children found at follow-up (78%). Mean birth weight was 2748 g (SD 534): 2777 g (SD 543) for males, and 2722 g (SD 524) for females. Low birth weight prevalence was 23.3% (352/1511), and very low birth weight prevalence 0.9% (14/1511). These figures are similar to those from the birth dataset. Table 3 shows that 35% of children under five had low weight for age, 47% low height for age, and 17% low weight for height. Boys appeared more likely to have low height for age than girls, but this difference was not significant (odds ratio 0.85; 95% CI 0.7, 1.04).

**Table 3:** Underweight, stunting and wasting in children at follow-up

	**Males**			**Females**			**All**		
	**n**	**N**	**(%)**	**n**	**N**	**(%)**	**n**	**N**	**(%)**
Weight for age z score									
< −2 SD	278	776	(35.8)	293	868	(33.8)	571	1644	(34.7)
< −3 SD	103	776	(13.3)	106	868	(12.2)	209	1644	(12.7)
Height for age z score									
< −2 SD	380	770	(49.4)	389	866	(44.9)	769	1636	(47.0)
< −3 SD	207	770	(26.9)	211	866	(24.4)	418	1636	(25.6)
Weight for height z score									
< −2 SD	136	764	(17.8)	137	853	(16.1)	273	1617	(16.9)
< −3 SD	41	764	(5.4)	46	853	(5.4)	87	1617	(5.4)

< −2 SD: more than 2 standard deviations below the median. The figures for children < −2 SD include those for < −3 SD.

**Figure 2:**
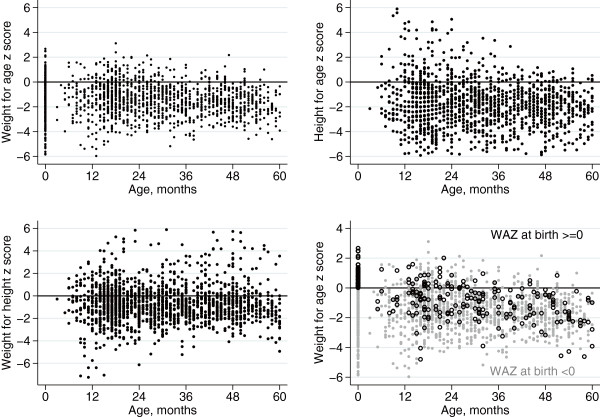
Scatterplots of weight for age, height for age, and weight for height z scores against child age.

**Figure 3:**
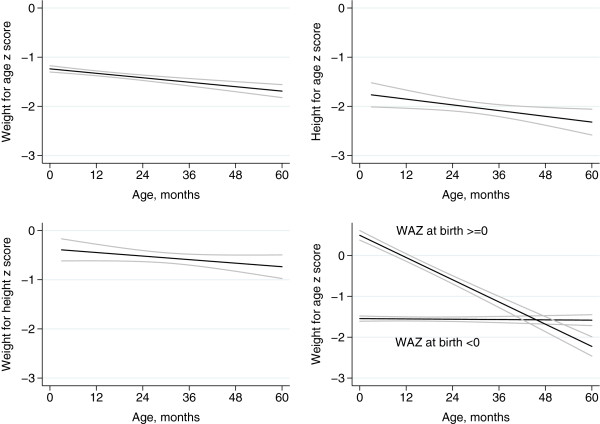
Fitted regression lines for weight for age, height for age, and weight for height z scores against child age, 95% confidence intervals.

Figures 2 and 3 show scatterplots and fitted regression lines for z scores by age. The scatterplot and fitted line for weight for age include z scores based on birth weight, taking age at birth as zero. Weight for age declined by −0.087 z scores per year of age (95% CI −0.119, -0.056) and height for age by −0.117 (−0.213, -0.021). Weight for height did not decline (−0.072; -0.160, 0.015). For a fourth scatterplot and fitted line, we divided the dataset into two strata: children born with weight for age z scores of zero or more (‘larger’), and of less than zero (‘smaller’). The decline in weight for age was almost entirely explained by 194 larger children, who showed a fall of −0.587 z scores per year (−0.651, -0.524); the 1097 smaller children showed no decline (0.006; -0.027, 0.039). Compared with mean scores at 36 months (−1.927, n = 62), 61% of faltering in HAZ score had occurred at 12 months of age (−1.170, n = 36), and 80% by 24 months (−1.536, n = 62).

## Discussion

Just under half of children born in 48 Mumbai slums were likely to develop stunting, although their malnutrition may not have been obvious because they were generally not overtly thin for their stature. Socioeconomic status was a clear determinant, larger infants tended to ‘catch down’, and the pattern may have been set well before birth.

Our estimate of mean birth weight (2736 g) accords with others from India, but was almost certainly an overestimate, since 14% of birth weights were unavailable and disproportionately represented infants from families of lower socioeconomic status. The prevalence of low birth weight (22%) was also probably an underestimate and showed data heaping characteristic of institutional records. About 21% of infants were born with low weight on the basis of z scores, and the associations were unsurprising: poverty [7,18], younger maternal age, less education, lower parity [19,20], multiple pregnancy, and preterm delivery. We also found positive associations with rest, food intake, and consumption of iron supplements during pregnancy, and with uptake of antenatal care.

At follow-up, 35% of children were underweight, 17% wasted, and 47% stunted. Stunting appeared to be established early and the subsequent decline in height for age was limited. Weight for height was not as compromised, suggesting that children followed a stunted trajectory established early (probably before birth). Infants born with higher weight for age z scores showed a downward trajectory.

Our study has three obvious limitations. We did not have measurements of length at birth, the number of data points in the first year was small, and the data represented a single follow-up for each child. This means that the impression of linearity in the trends in z score change might be the result of sampling a long period (from birth to 60 months). A fall in the first six months, for example, might not be seen. Or a series of curves – each belonging to a single infant and plotted prospectively – might superimpose to give the impression of a straight line. We accept these possibilities. It would be better to follow infants serially over the first year of life in more detail, and this we intend to do. It would also be good to have accurate measurements of length at birth, although the procedure requires training and, usually, bespoke studies.

Three issues make us unwilling to reject our hypotheses, however. First, our general anthropometric findings were similar to those of the most recent NFHS, and particularly its subsample from Mumbai slums [5]. Second, we were able to link follow-up weights with birth weights, and there should be an intuitive connection between size at birth and later size.

The third issue is the similarity of our findings with those of a recent analysis of two NFHS datasets [21]. Mamidi and colleagues suggested that 44% of total faltering in weight for age had taken place by the time of birth, and 71% by six months of age. There was no further faltering from six to twelve months, and faltering was complete by 24 months. For height for age, 65% of the faltering in mean HAZ seen by 36 months of age had occurred by 12 months, and 96% by 24 months. Analogous figures from our study were 61% and 80%, and support the idea that much of growth faltering is explained by faltering in height for age, rather than by wasting. We share the authors’ concern that much of the “… total growth faltering in India has already taken place at birth.”

It could be argued that early stunting is one way to interpret the findings, but that the children may be constitutionally smaller and have different body habitus from children in other settings [22]. There is a longstanding debate about whether growth standards should be locally tailored or reflect global distributions. Given discussions on the developmental origins of health and disease, our current opinion is that short stature in Indian children and adults is largely explained by a combination of macro- and micro-environmental factors whose effects are to some degree trans-generational (and possibly inherited epigenetically). So long as international standards are applied as benchmarks, high proportions of Indian children will continue to be classified as underweight and stunted, and our inferences should be considered within this framework.

We think that our findings raise two major issues. First, given current concerns about body composition and longer-term disease [22], the benefits of supplementing the diets of children at later ages are unclear [23]. Increased dietary intake is unlikely to change height trajectory substantially, and there are potential risks to increasing weight for height in terms of later glycemic control. The best that can be said for the existing Integrated Child Development Services is that they begin too late (at three years, at least for food supplementation) to have an optimal effect, and we agree with current calls to focus on a younger age-group [3,24,25]. Pregnant women are also eligible for support, but uptake is limited. Second, weaning has often been seen as a critical point for input, with the idea that it is associated with a change in nutritional trajectory [26]. It is, of course, possible that the growth faltering of children in urban slums has a different pattern to that in rural areas, but we think that this assumption requires further examination.

Interestingly, the overall steady downward progress in weight for age involved relative maintenance of z score in infants born smaller, and relatively greater decline in infants born larger. It is worrying that these larger infants appear to track downwards. One possibility is that this represents regression to the mean. Another is that environmental influences conspire against the healthy growth of children in Mumbai’s slums. This is a sobering thought. Whatever our eventual understanding, we recommend a focus of research on potential intervention in the first 1000 days. Of perhaps greater importance in the longer term is to break the cycle by focusing on nutrition and education for young girls.

## Competing interest

None of the authors has a conflict of interest.

## Authors’ contributions

SD and UB managed field data collection. SD and DO did the quantitative analysis. NSM was the director of the project from which the data derive, GA was technical advisor to the study, and AF had oversight of programme activities. DO wrote the first manuscript draft and was responsible for subsequent collation of inputs and redrafting. DO will act as guarantor. All authors read and approved the final manuscript.
